# Thermally Tunable Ultra-wideband Metamaterial Absorbers based on Three-dimensional Water-substrate construction

**DOI:** 10.1038/s41598-018-22163-6

**Published:** 2018-03-13

**Authors:** Yang Shen, Jieqiu Zhang, Yongqiang Pang, Lin Zheng, Jiafu Wang, Hua Ma, Shaobo Qu

**Affiliations:** 1 College of Science, Air Force Engineering University, Xi’an, Shaanxi 710051 People’s Republic of China; 2 School of Electronics and Information Engineering, Xi’an Jiaotong University, Xi’an, Shaanxi 710049 People’s Republic of China

## Abstract

Distilled water has frequency dispersive characteristic and high value of imaginary part in permittivity, which can be seen as a good candidate of broadband metamaterial absorbers(MAs) in microwave. Here, an interesting idea based on the combination of water-substrate and metallic metamaterial in the three-dimensional construction is proposed, which can achieve outstanding broadband absorption. As a proof, the distilled water is filled into the dielectric reservoir as ultra-thin water-substrate, and then the water-substrates are arranged on the metal backplane periodically as three-dimensional water-substrate array(TWA). Simulation shows that the TWA achieves broadband absorption with the efficiency more than 90% from 8.3 to 21.0 GHz. Then, the trigonal metallic fishbone structure is introduced here between the water-substrate and the dielectric reservoir periodically as three-dimensional water-substrate metamaterial absorber(TWMA). The proposed TWMA could achieve ultra-broadband absorption from 2.6 to 16.8 GHz, which has increase by 64.8% in relative absorption bandwidth. Meanwhile, due to the participation of distilled water, the thermally tunable property also deserves to be discussed here. In view of the outstanding performance, it is worth to expect a wide range of applications to emerge inspired from the proposed construction.

## Introduction

Metamaterials, stemming from a pursuit of exotic electromagnetic property, have been equipped with more diversified performances for light modulation, such as electromagnetic wave absorption, abnormal refraction, and destructive scattering. With the continuous innovation in metamaterials, a lot of well-chosen materials were introduced here for the construction of new artificial media, such as all-dielectric ceramic^1–4^, phase-change material^5–8^, and graphene^8–12^. The according metamaterial would achieve more improvement as well as the desired electromagnetic property, such as high-temperature-tolerance character, thermally switchable property, and electrically reconfigurable performance.

For the electromagnetic wave absorption, the first MA based on the three-layered construction of metal-dielectric-metal was proposed in microwave frequency^13^. The strong resonance inspired by the metallic electric resonator worked together with the suitable loss from Fr4 substrate, which contributed to near perfect absorption at a certain frequency. Then, with the emerging of metamaterials, single-, dual-, and multi-band absorbers can be flexibly gained^14–19^. However, it should be pointed out that the highly effective absorption was always accompanied with narrow band characteristic, which has always been an obstacle for the application requirements. To acquire broadband MA, the spatial arrangements of multi-unit with contiguous resonances were firstly proposed^20–26^. The varied MA units can be assembled together not only on the same plane^20–23^ but also along incident wave vector^24–26^. However, the aforementioned MAs effectively broadened the absorption bandwidth at the sacrifice of high absorbing efficiency, ultra-thin thickness or lightweight characteristic. Meanwhile, frequency dispersive materials can also be designed as resonators^27,28^ or substrates^29–31^ in MAs, which also contributed to the broadband absorption. However, the fabricated frequency dispersive materials are not easy to meet the required values of the effective permittivity/permeability.

Recently, due to the frequency dispersive characteristic and high value of imaginary part in permittivity, distilled water was seen as a good candidate in MA for broadband microwave absorption. On the one hand, distilled water can be filled into the dielectric reservoir as all-dielectric resonator^32–35^. After optimization, the periodic water-based resonator array backed with metal backplane can exhibit broadband absorption in microwave frequency. On the other hand, distilled water can also be used as frequency dispersive substrate^36^. When combining with the patterned metallic layer on the surface and the metal backplane on the bottom, the three-layered water-substrate MAs were easy to achieve highly effective and broadband absorption. Moreover, due to the participation of distilled water, the water-based MAs were also equipped with mechanically and thermally tunable performance^32^. Therefore, distilled water can be seen as a good candidate for broadband absorption in microwave frequency. However, the broadband absorption of water-based MAs in the literature almost concentrated on the frequency band above C-band, and the further improvement of lower-frequency absorption, especially for S-band, was still a challenge.

In this paper, we develop distilled water as substrate array on the metal backplane and then loading metallic metamaterials to achieve ultra-wideband absorption covering the frequencies from S-band to Ku-band. As a proof, the distilled water is firstly filled into the dielectric reservoir as ultra-thin water-substrate, and the water-substrates are arranged on the metal backplane periodically as TWA. Then, the trigonal metallic fishbone structure is introduced here between water-substrate and dielectric reservoir periodically as TWMA. Simulation and experimental result demonstrate that the proposed TWMA achieves ultra-broadband absorption from 2.6 to 16.8 GHz, which had increase of 64.8% in relative absorption bandwidth compared to the TWA. Moreover, due to the participation of the distilled water, the thermally tunable property is also discussed here. Due to its outstanding performance, it is worth to expect a wide range of applications to emerge inspired from the proposed attempt.

## Results

### Three-dimensional water-substrate array

**Figure 1:**
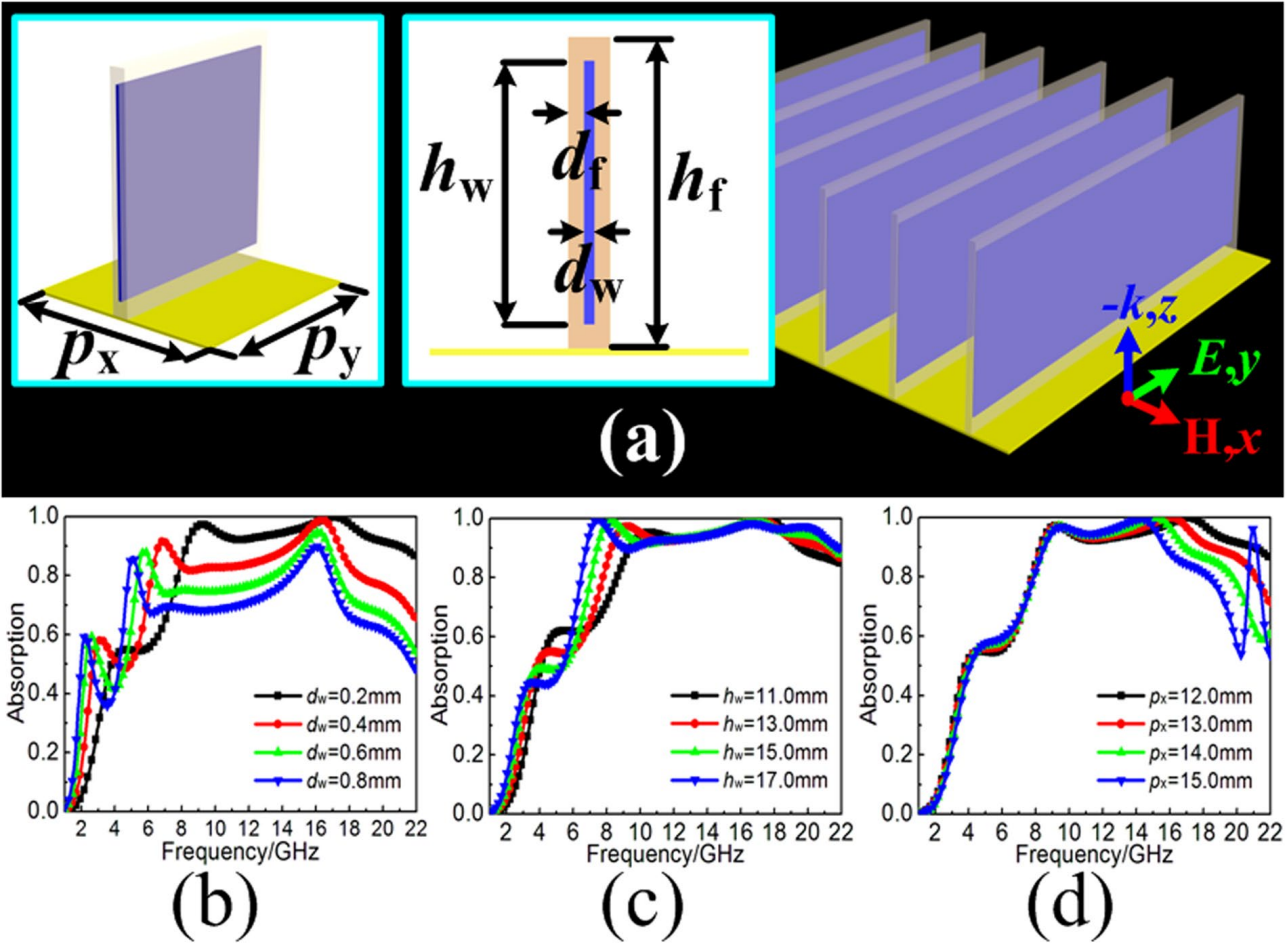
(**a**) Structure diagram and perspective view of the TWA. Simulated absorption spectra by varying: (**b**) the thickness *d*_w_ of water-substrate, (**c**) the height *h*_w_ of water-substrate, (**d**) the arranged dimension *P*_x_ of adjacent water-substrates.

In fact, the permittivity of the distilled water can be described by the Debye formula under the certain environmental temperature of *T*_*water*_ as follows:

1 $$\varepsilon (\omega ,{T}_{water})={\varepsilon }_{\infty }({T}_{water})+\frac{{\varepsilon }_{0}({T}_{water})-{\varepsilon }_{\infty }({T}_{water})}{1-i\omega \tau ({T}_{water})}$$

where *ε*_∞_ and *ε*_0_ are the optical permittivity and static permittivity, *τ* is the rotational relaxation time, which has been detailed discussed in^37^. The permittivity of distilled water was frequency dispersive as well as high value of imaginary part in permittivity, which contributes to the highly effective absorption in microwave frequency. However, the mismatch of the impedance during a broad frequency region has always been the obstacle. Thus, water-based MAs consist of subwavelength resonator could be the effective way to achieve broadband absorption. Here, we fill the distilled water into the dielectric reservoir as ultra-thin rectangle water-substrate, as the insets shown in Fig. 1(a). The height and thickness of the dielectric reservoir are *h*_f_ and *d*_f_, respectively. While the height and thickness of the filled-water are *h*_w_ and *d*_w_. The rectangle water-substrates are arranged on the metal plate periodically with the dimension of *P*_x_. The permittivity of distilled water is calculated by the theoretical model under the practical condition of room temperature(15 °C) and a standard atmospheric pressure. The dielectric reservoirs are constructed by the Fr4 substrate with the permittivity and loss tangent are 4.3 and 0.025, respectively. The metal used here is copper with the conductivity of 5.8 × 10^7^s·m^−1^. In the simulation, the electric field of incidence is required to along *y*-axis to ensure the optimal broadband absorption due to the polarization-dependence of the proposed array. The absorptive efficiency of the TWA under normal incidence can be defined as *A*(ω) = 1 − *R*(ω) − *T*(ω) = 1 − |S_21_|^2^ − |S_11_|^2^, where *A*(ω), |S_11_|^2^ and |S_21_|^2^ are the absorbance, reflectivity and transmissivity, respectively. Due to the metal backplane, the transmission (S_21_) is zero. Thus, the absorbance can be calculated by *A*(ω) = 1 − |S_11_|^2^ in this paper. Here, the important parameters are worth discussing in detail for the optimization of broadband absorption. Firstly, as shown in Fig. 1(b), the thickness *d*_w_ of distilled water directly determines the impedance of the proposed array which has a great influence on the absorbing efficiency during a broad frequency region. The highly effective absorption more than 90% can be achieved only for the thickness of distilled water less than 0.2 mm. Secondly, as shown in Fig. 1(c), the height *h*_w_ of the distilled water reflects the total height of absorbing medium which determines the relative absorption bandwidth. The absorption bandwidth can be appropriately broadened with the increased height of the absorbing medium. Lastly, as shown in Fig. 1(d), the arranged dimension *P*_x_ of water-substrate also directly determines the absorption peak of the high frequencies. The absorption peak will move to higher frequencies with the decreasing of arranged dimension *P*_x_. After optimization, the parameters of unit cell are given as follows: *P*_x_ = 12.0 mm, *P*_y_ = 12.0 mm, *h*_f_ = 15.0 mm, *d*_f_ = 0.4 mm, *h*_w_ = 13.0 mm, and *d*_w_ = 0.2 mm, the simulation shows that the TWA can achieve broadband absorption with the efficiency more than 90% in the frequency band of 8.3–21.0 GHz.

**Figure 2:**
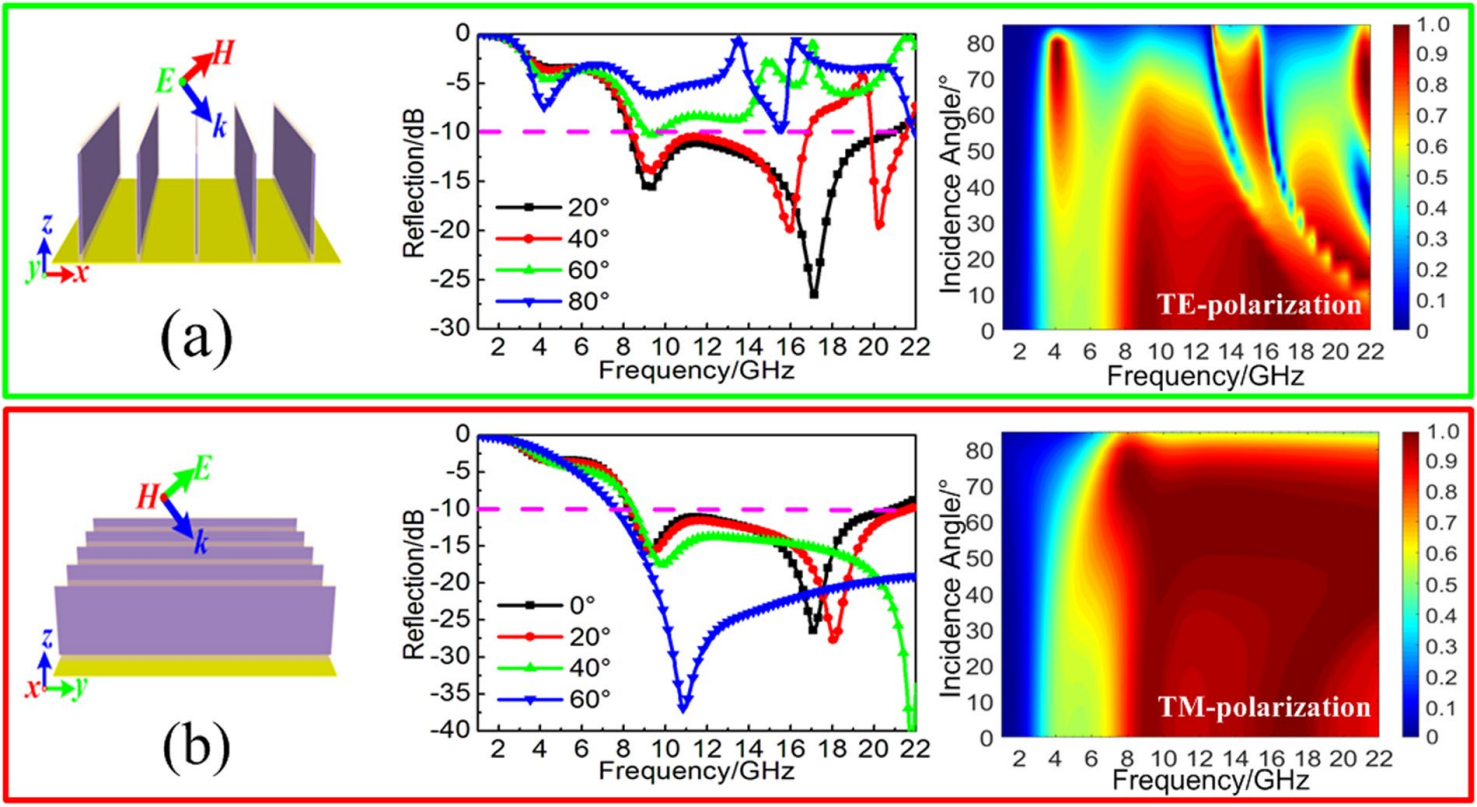
Simulated reflectivity spectra and absorption spectra of the TWA under the different oblique incident waves of (**a**) TE polarization and (**b**) TM polarization.

In addition, we also discuss the wide-angle absorption performance of the proposed TWA under the oblique incident wave of transverse electric(TE) and transverse magnetic(TM) polarization, respectively. As the insert shown in Fig. 2(a), for the oblique incident wave of TE polarization, the electric field is always along *y*-axis while the magnetic field changes with the wave vector. With the increasing of incident angles, not only will the absorbing efficiency gradually decrease during the operating frequencies region, but the continuous broadband absorption band will also disappear due to the grating lobe effect. Meanwhile, as the insert shown in Fig. 2(b), for the oblique incident wave of TM polarization, the magnetic field is always along *x*-axis and the electric field changes with the wave vector. With the increasing of incident angles, there has obvious enhancement of broadband absorption. For the incident angle below 75°, both of the absorbing efficiency and the absorption bandwidth are gradually improved with the increasing of incident angle. Compared with the other MAs in the literature, the angle-dependent absorption performance is almost consistent. The underlying mechanism accounts for the difference is rotation out of plane of the magnetic field for TE wave and thus the effective magnetic response of the metamaterial unit cell is reduced, which leads to a lowering of the absorption^38^. In contrast, the incident magnetic field is always in the plane for the TM wave while the electric field gradually rotates out of plane. The effective electric response of the metamaterial unit cell is accordingly reduced, which contributes to the enhancement of absorption for oblique TM wave within limits.

**Figure 3:**
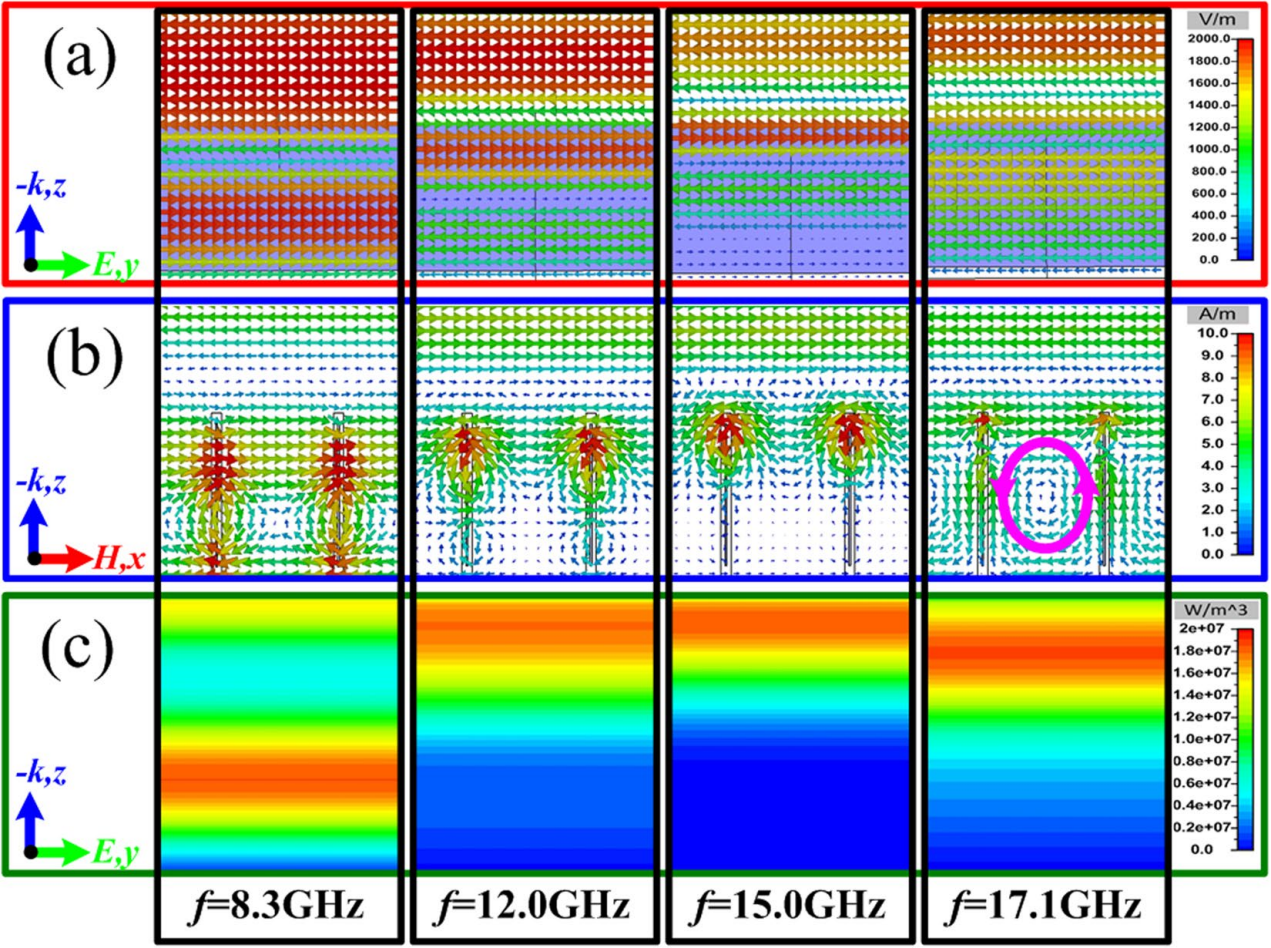
(**a**) The electric field intensity distribution from the view of *x*-axis. (**b**) The magnetic field intensity distribution from the view of *y*-axis. (**c**) The energy loss distribution form the view of *x*-axis of the TWA at different frequencies of 8.3 GHz, 12.0 GHz, 15.0 GHz, and 17.1 GHz.

To analyze the absorption principle, the electric field intensity distribution, the magnetic field intensity distribution and the energy loss distribution of the TWA at different frequencies of 8.3 GHz, 12.0 GHz, 15.0 GHz and 17.1 GHz are given in Fig. 3(a)–(c), respectively. From the field distributions at the frequency of 8.3 GHz, 12.0 GHz and 15.0 GHz, there is obvious standing-wave effect with the field enhancement on the surface of the water-substrate at different heights. Thus, the multi-standing-wave is inspired by the TWA in a broad frequency band. For the high frequency of 17.1 GHz, the wavelength of incidence has similar dimension to the district between the adjacent water-substrates, and the magnetic field in the district comes into being an obvious circulation with the field enhancement as the pink line marked in Fig. 3(b). The arranged dimension *P*_x_ between the adjacent water-substrates directly determines the operating frequency, which can be seen as structural resonance. The multi-standing-wave and the structural resonance exited by the TWA overlap together during a broad frequency band. When loading enough loss from the distilled water, the proposed TWA will exhibit highly effective and broadband absorption. However, the multi-resonance inspired by the TWA is still limited, the further improvement of broadband absorption, especially for the lower-frequency absorption, is still far from our expectation.

### Three-dimensional water-substrate MA

**Figure 4:**
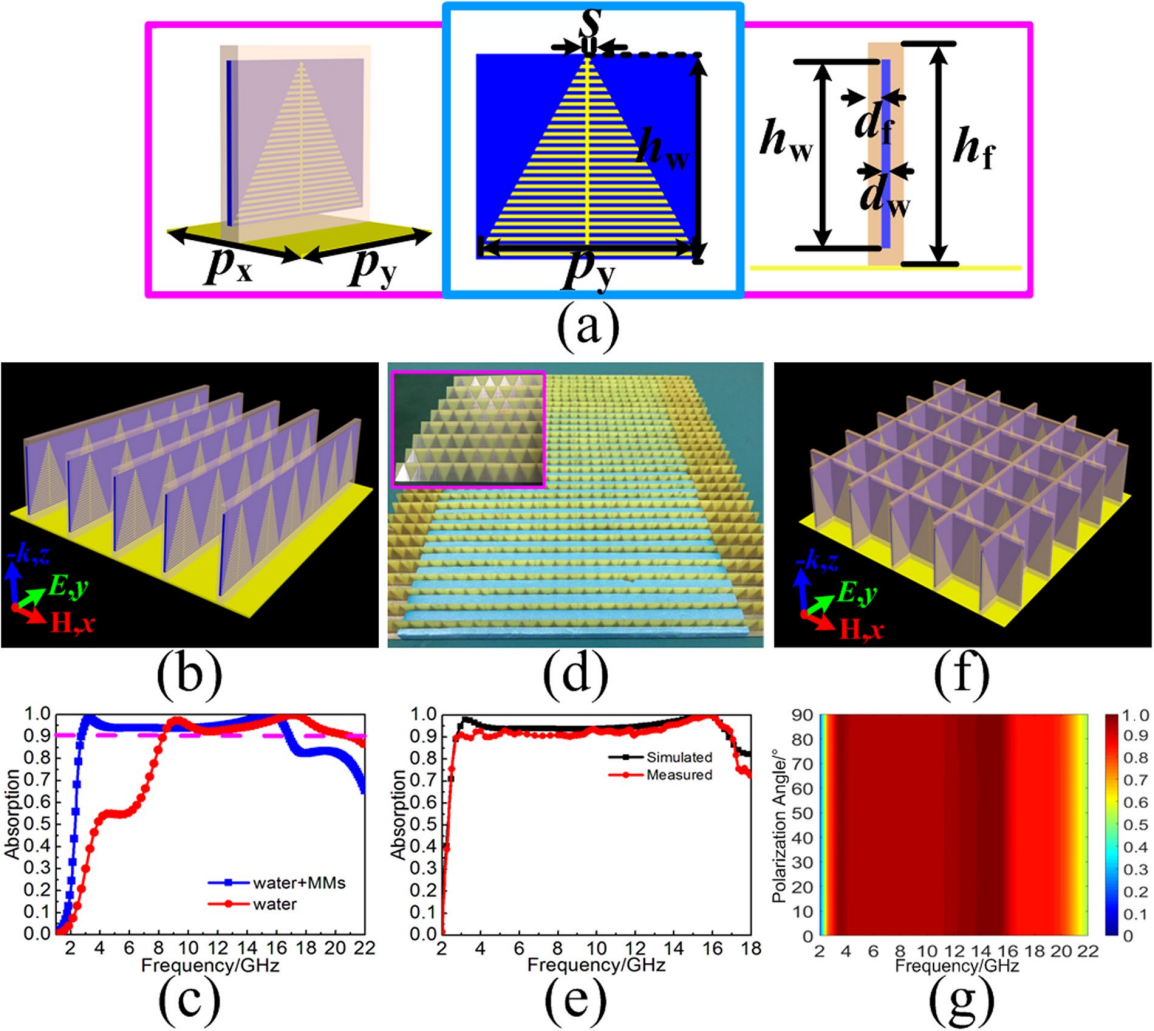
(**a**) Structure diagram of the TWMA. (**b**) Perspective view of the TWMA. (**c**) Comparison of simulated absorption spectra for the TWA loading with and without trigonal metallic fishbone structure. (**d**) Fabricated sample of the TWMA. (**e**) Comparison of the simulated and measured absorption spectra for the TWMA. (**f**) Perspective view of the TWMA with polarization-independent construction. (**g**) The absorption spectra of TWMA with different polarized wave under normal incidences.

**Figure 5:**
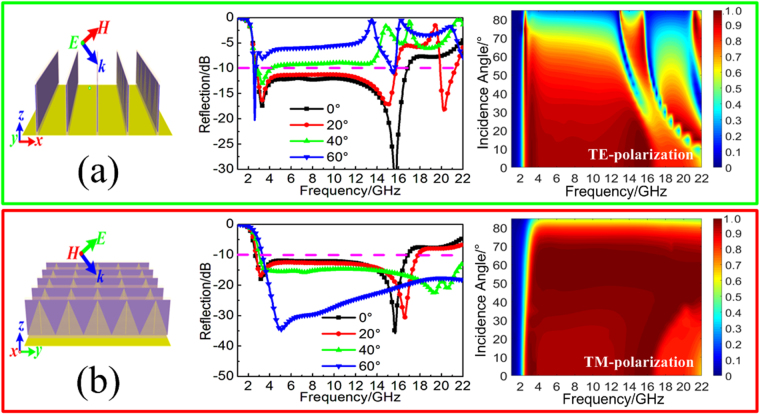
Simulated reflectivity spectra and absorption spectra of the TWMA with the different oblique incident waves of (**a**) TE polarization and (**b**) TM polarization.

Based on the aforementioned TWA, we attempted to load metallic pattern on the side of water substrate for further improvement of broadband absorption, especially for low frequencies. To make full use of the loss from the distilled water, the fishbone structure should be close to the distilled water as soon as possible. As the structure diagram shown in Fig. 4(a), the trigonal metallic fishbone is adhered between the distilled water and the dielectric reservoir periodically with the arranged dimension of *P*_y_ along *y*-axis. To be specific, the metallic line with the same width of *s* constructs the proposed metallic fishbone. The multiple parallel metallic strips with gradually varied length are arranged with the dimension of 2·*s*, and then, they are bunched together by the middle strip spine. The profile of the fishbone structure is isosceles triangle with the lower side of *P*_y_ and height of *h*. Adopting the technology of standard PCB photolithography, the average thickness of the metallic fishbone is about 0.017 mm. After arranging the proposed MA unit cell with the arranged dimension of *P*_x_ along the *x*-axis, the TWMA is achieve in Fig. 4(b). In the simulation, the electric field is still set along *y*-axis direction due to the polarization dependent property. When giving the parameters of the proposed MA unit cell as follows: *P*_x_ = 12 mm, *P*_y_ = 12 mm, *h*_f_ = 15.0 mm, *d*_f_ = 0.4 mm, *h*_w_ = 13.0 mm, *d*_w_ = 0.2 mm, and *s* = 0.2 mm, the simulated result shows that the TWMA can achieve ultra-wideband absorption with the efficiency over 90% from 2.8 to 16.8 GHz. Compared with the TWA, the TWMA has increase by 64.8% in relative absorption bandwidth, which is shown in Fig. 4(c). Then, the further experimental demonstration of the TWMA is also performed. As the fabricated sample shown in Fig. 4(d), the dimension of the fabricated sample is 360 × 360 mm^2^, which consists 30 units of water-substrates arranged long *x*-axis. Each water-substrate is adhered with 30 units of trigonal metallic fishbone arranged long *y*-axis. In the fabrication and measurement process, the distilled water used here are always under the practical condition of the room temperature of 15 °C and a standard atmospheric pressure. The experimental demonstration of fabricated sample is performed by the arch measurement system in a microwave anechoic chamber. The system is based on an Agilent E8363B network analyzer with three pairs of broadband antenna horns respectively working in the frequency bands of 2–8, 8–12, and 12–18 GHz. As shown in Fig. 4(e), the measured result is in a good agreement with the simulation when taking into account of the fabrication roughness as well as the measurement errors. Meanwhile, to achieve polarization-independent absorption, the proposed MA unit cell is rolling as a square grid in Fig. 4(f). The simulation shows that the TWMA can achieve ultra-wideband absorption with efficiency more than 90% from 3.1 to 16.2 GHz under normal incidence, and the polarization-independent absorption performance is also proved in Fig. 4(g). However, due to the relatively complex construction, the fabrication of the polarization-independent TWMA needs the help of 3D printing technology. In addition, the wide-angle absorption performance for the proposed TWMA is also discussed here. Figure 5(a) shows the simulated reflectivity spectra and absorption spectra of the TWMA under different oblique incident wave of TE polarization. Although the influence of grating lobe effect still exits at high frequencies, the wide-angle absorption is obviously improved within the angle of 60°. While for the oblique incident wave of TM polarization in Fig. 5(b), both of the absorbing efficiency and the absorption bandwidth are gradually improved with the increasing of incident angle until 80°. In contrast, the wide-angle absorption performance for the proposed TWMA is obvious improved compared to the TWA. Thus, loading trigonal metallic fishbone structure to the TWA not only contributes the achievement of ultra-wideband absorption, but it also improves the wide-angle absorption performance.

**Figure 6:**
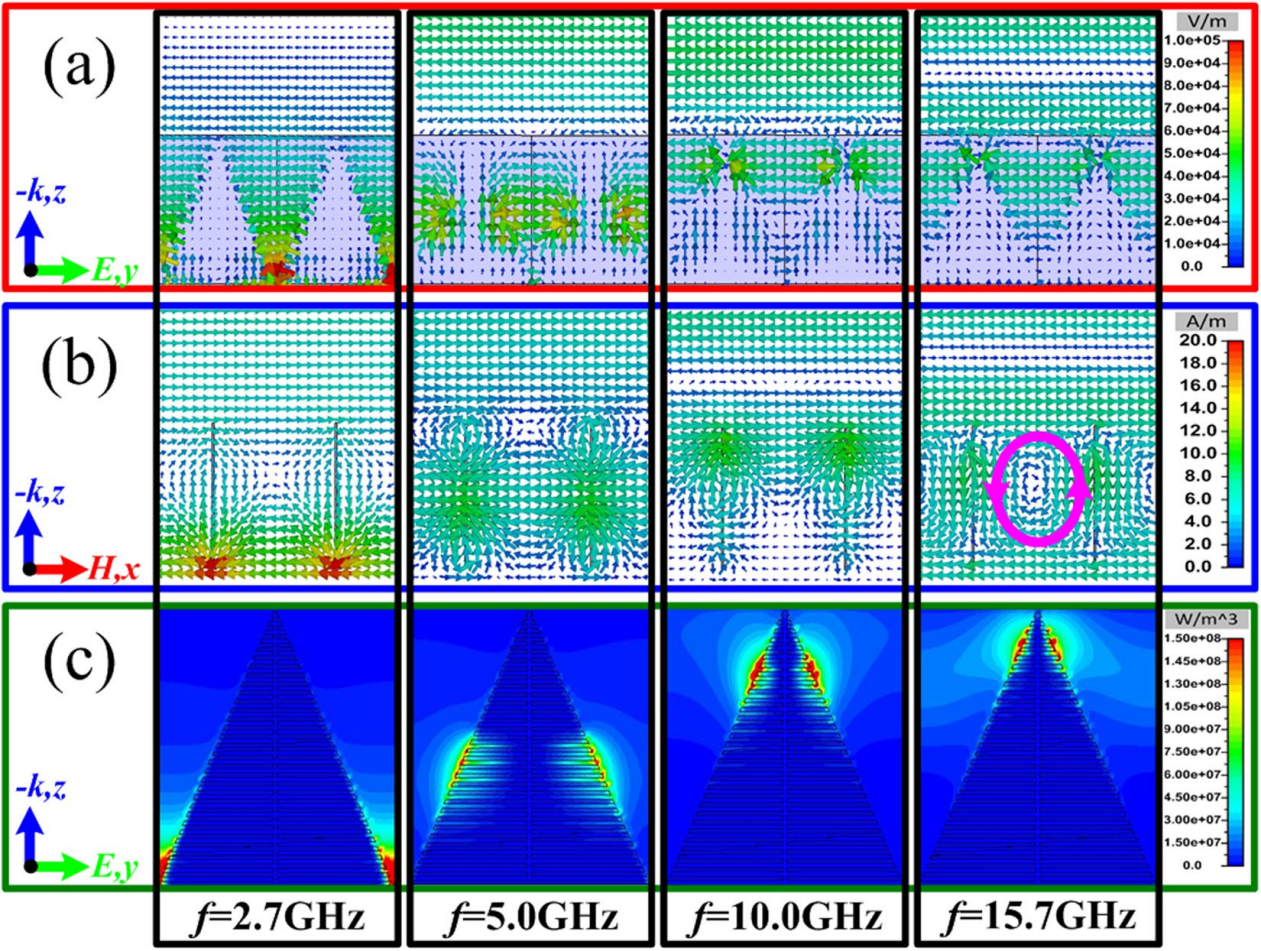
(**a**) The electric field intensity distribution from the view of *x*-axis. (**b**) The magnetic field intensity distribution from the view of *y*-axis. (**c**) The energy loss distribution form the view of *x*-axis of the TWMA at different frequencies of 2.7 GHz, 5.0 GHz, 10.0 GHz, and 15.7 GHz.

To intuitively understand the operating principle, the electric field intensity distribution, the magnetic field intensity distribution and the energy loss distribution of the TWMA at different frequencies of 2.7 GHz, 5.0 GHz and 10.0 GHz are given in Fig. 6(a)–(c), respectively. For the frequencies of 2.7 GHz, 5.0 GHz and 10.0 GHz, the fields are obvious enhanced at different heights on the interface between the metallic fishbone structure and water-substrate. The energy loss is also dissipated at the corresponding positions. Thus, the trigonal metallic fishbone structure on the substrate can be seen as a combination of gradually varied MA, which excites the multi-resonance during an ultra-wide frequency band. While for the high frequency of 15.7 GHz, the circulation of magnetic field is also excited between the adjacent water-substrates, which can be seen as the structural resonance marked by the pink line in Fig. 6(b). From the aforementioned field distribution, the excited multi-resonance of metallic fishbone structure is accompanied with the obvious field enhancement during an ultra-wide frequency region. Then, combined with the enough loss from the distilled water, the TWMA can easily achieve highly effective electromagnetic wave absorption in an ultra-wide frequency region.

**Figure 7:**
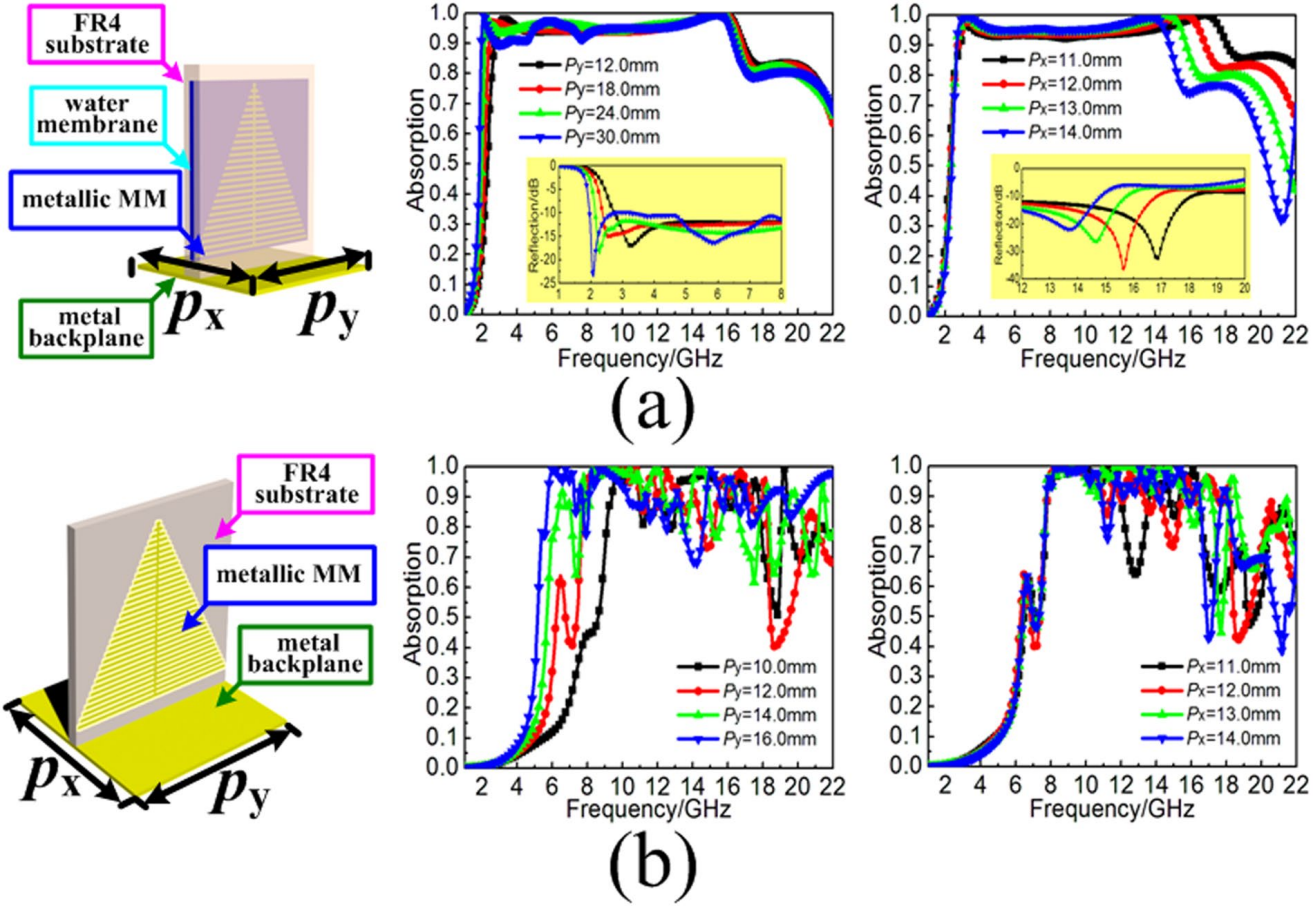
(**a**) Simulated absorption spectra for the TWMA by varying: the arranged dimension *P*_y_ of metallic fishbone structure along *y*-axis and the arranged dimension *P*_x_ of MA unit cell along *x*-axis. (**b**) Simulated absorption spectra for the three-dimensional MA by varying: the arranged dimension *P*_y_ of metallic fishbone structure along *y*-axis and the arranged dimension *P*_x_ of MA unit cell along *x*-axis.

To demonstrate that water-substrate plays equally important role for the improvement of broadband absorption, the water-substrate of the proposed MA is replaced with the Fr4 substrate as a comparison. For the TWMA, the parameter of *P*_y_ reflects the arranged dimension of triangle metallic fishbone as well as the length of the longest fishbone strip along *y*-axis. As the inserts shown in Fig. 7(a), with the increasing of *P*_y_ from 12.0 to 30.0 mm, the lower boundary frequency of ultra-wideband absorption with the reflection below −10 dB would further expand to lower frequency from 2.8 to 2.0 GHz. While the *P*_x_ reflects the arranged dimension between the adjacent MA unit cell along *x*-axis. With the decreasing of *P*_x_ from 14.0 to 11.0 mm, the upper boundary frequency of ultra-wideband absorption with the reflection below −10dB would further expand to higher frequencies from 14.8 to 18.0 GHz. From the absorption spectra in Fig. 7(a), it is satisfying that the continuous and highly effective absorption performance is almost unaffected during the parameter optimization. By contrast, when replacing the water-substrate with the Fr4 substrate, the continuous and highly effective broadband absorption is greatly influenced. As shown in Fig. 7(b), with the change of parameters *P*_x_ and *P*_y_, the MA is still unable to achieve the continuous ultra-wideband absorption as well as high absorbing efficiency. Thus, it can be concluded that both of the triangle metallic fishbone structure and the water-substrate play indispensable role in TWMA for the achievement of ultra-wideband absorption. Furthermore, we also compare the proposed TWMA against several similar designs taken from the literature in terms of absorption bandwidth, thickness, and relative absorption bandwidth in Table 1 to discuss their broadband absorption performance. Compared with other broadband MAs, the proposed TWMA have obvious advantage in the achievement of ultra-wideband absorption, especially for lower frequency absorption.

**Table 1:** Broadband absorption performance comparison.

Reference	Absorption bandwidth(GHz)	Thickness(mm)	Relative absorption bandwidth
^21^	10.0–12.2	0.8	19.8%
^24^	8.1–14.0	5.0	53.1%
^29^	6.0–18.0	2.0	100.0%
^35^	8.1–22.9	5.6	95.5%
^36^	6.2–19.0	3.5	101.6%
This Work	2.8–16.8	15.0	142.9%

### Thermally tunable broadband absorption

**Figure 8:**
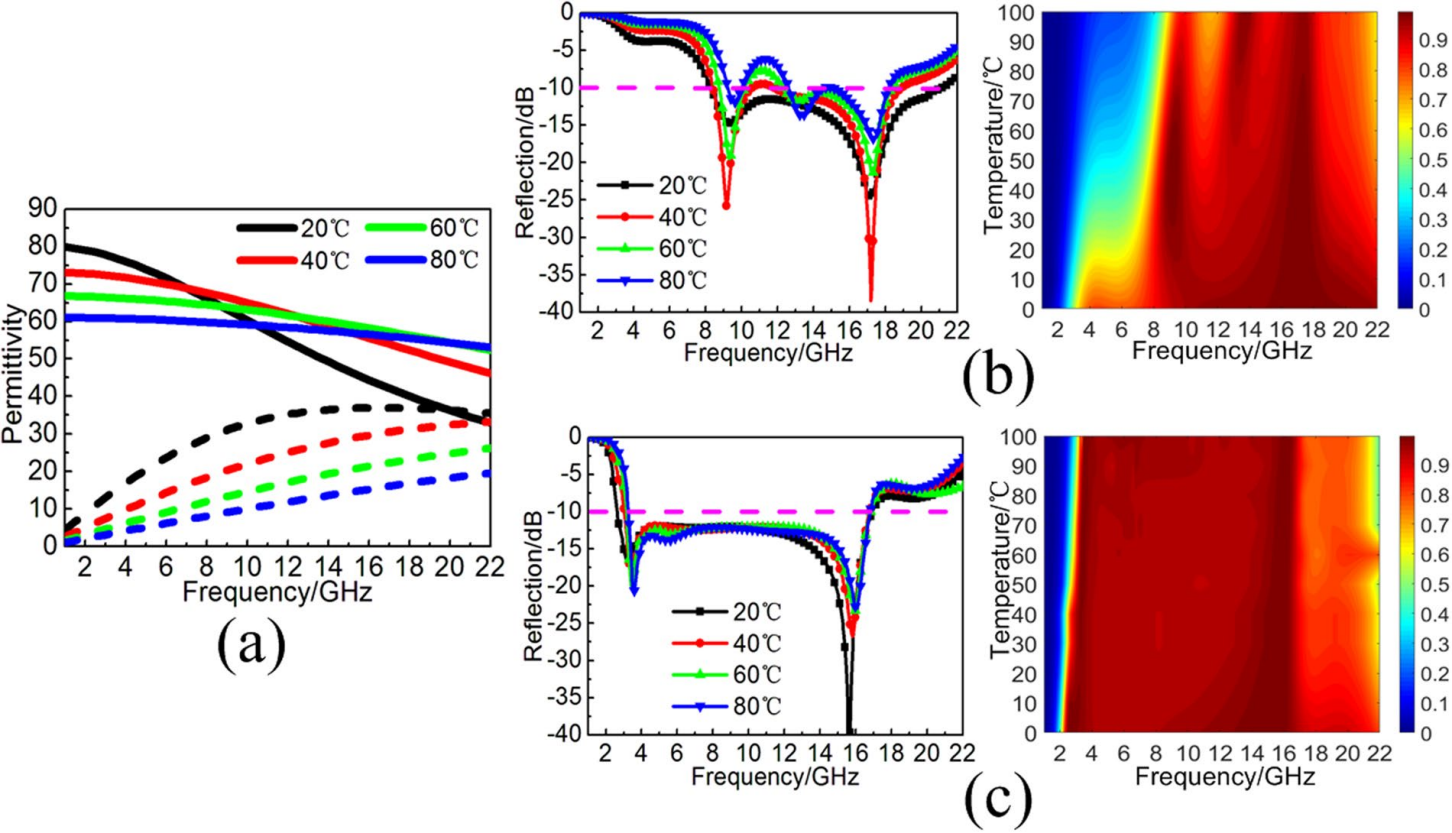
(**a**) Permittivity of distilled water calculated by the Debye formula under different environmental temperature of 20 °C, 40 °C, 60 °C, and 80 °C. (**b**) Simulated reflectivity spectra and absorption spectra of the TWA under different environmental temperature. (**c**) Simulated reflectivity spectra and absorption spectra of the TWMA under different environmental temperature.

The aforementioned discussion about the TWMA just considers the practical condition of room temperature (15 °C) and a standard atmospheric pressure. In fact, it is well known that the permittivity of distilled water changes obviously with the environmental temperature, which can be seen as thermally tunable property shown in Fig. 8(a). Thus, the varied permittivity of distilled water will also have influence on the absorption performance in TWA and TWMA. Figure 8(b) shows the simulated reflectivity spectra and absorption spectra of the TWA under different environmental temperature. With the increasing of environmental temperature from 0 to 100 °C, not only will the absorption bandwidth be gradually narrowed, but the absorbing efficiency will also be reduced accordingly. Thus, the broadband absorption of the TWA is obviously tunable by the environmental temperature. Meanwhile, Fig. 8(c) shows the simulated reflectivity spectra and absorption spectra of the TWMA under different environmental temperature. With the increasing of environmental temperature from 0 to 100 °C, the ultra-wide absorption bandwidth with efficiency more than 90% decreased from 2.3–16.8 GHz to 3.4–16.8 GHz. The obvious difference between the two MAs mainly results from the different absorbing mechanism. As we know, the electromagnetic power absorption in a non-magnetic medium is mainly determined by losses as well as electric filed strength, following the relation

2 $${P}_{abs}=\frac{1}{2}(\omega \varepsilon ^{\prime\prime} +\sigma ){|E|}^{2}$$

where *ω* is the angular frequency, *ε*″ is the imaginary part of permittivity, *σ* is the conductivity and *E* is the total electric field. Compared the electric field intensity distribution in Figs 3(a) and 6(a), the locally electric field inspired by the subwavelength structure of metallic fishbone is much higher than the electric field of multi-standing-wave in the water-substrate array. Thus, the TWMA is easier to achieve highly effective absorption performance during a wide frequency band. In other word, according to the aforementioned formula, the higher electric field *E* and the conductivity *σ* make the imaginary part *ε*″ of the distilled water play a smaller role in electromagnetic wave absorption. Based on aforementioned discussion, the thermally tunable absorption of the TWA must be more active than the TWMA. Therefore, broadband MA based on the distilled water can provide diversified forms of thermally tunable absorption performance. On the hand, MAs merely consist of distilled water can exhibit actively tunable broadband absorption. On the other hand, MAs based on the combination of distilled water and metallic metamaterials can also exhibit temperature-independent broadband absorption.

## Conclusions

In conclusion, distilled water has been demonstrated as a good candidate for the achievement of thermally tunable ultra-wideband MA in microwave frequency. As a proof, we fill the distilled water into the dielectric reservoir as ultra-thin water-substrate, and then the water-substrates are arranged on the metal backplane periodically as TWA. The TWA achieves broadband absorption with the efficiency more than 90% from 8.3 to 21.0 GHz. To further improve the absorption bandwidth, especially for lower-frequency absorption, the trigonal metallic fishbone structure is introduced between the water-substrate and the dielectric reservoir periodically as TWMA. Simulation and experimental result demonstrate that the TWMA achieves ultra-wideband absorption with the efficiency more than 90% from 2.6 to 16.8 GHz, which has increase by 64.8% in relative absorption bandwidth. Moreover, due to the participation of the distilled water, the thermally tunable property of the proposed MA is also discussed here. Due to its outstanding performance, it is worth to expect several applications inspired from the proposed construction in the future.

## Method

### Simulations

Electromagnetic simulations are performed using a commercially available software package, CST Microwave Studio. The *S* parameters are simulated using the frequency domain solver and the field distributions(electric field intensity, magnetic field intensity, and energy loss) are monitored simultaneously. In the simulation, unit cell boundary conditions in the *x* and *y* directions and open at space boundary conditions in the *z* direction are used.

### Fabrication

In fact, the desired method of the fabricated dielectric reservoir should be three-dimensional printing technology. However, considering the difficulties that the trigonal metallic fishbone structure is adhered to the intine of the dielectric reservoir and the distilled water should be filled into the fabricated reservoirs, the three-dimensional printing technology has temporary incompetence. Here, we attempt to construct the fabricated sample using the PCB circuit board step by step. Firstly, Fr4 substrates with the thickness of 0.4 mm printed with or without trigonal metallic fishbone structure are glued together by the PET gaskets in the middle. The gasket is small enough which provides the space with the thickness of 0.2 mm for distilled water. The epoxy resin is used here to close off the three sides of the space. Then, distilled water is filled into the aforementioned dielectric reservoir and then using the epoxy resin to close off the last side. In the fabrication of the proposed TWMA unit cell, it is noted that one of the Fr4 substrate printed with trigonal metallic fishbone structure should face the inside space. The water-substrates printed with the trigonal metallic fishbone structure is firstly fabricated and then assembled together on the metal backplane. The dimension of the fabricated sample is 360 × 360 mm^2^, which consists 30 units of water-substrates arranged long *x*-axis. Each water-substrate is adhered with 30 units of trigonal metallic fishbone structures arranged long *y*-axis.

### Measurements

The experimental study of the fabricated sample is performed by the arch measurement system in a microwave anechoic chamber. The system is based on an Agilent E8363B network analyzer with three pairs of broadband antenna horns, respectively, working in the frequency bands of 2–8 GHz, 8–12 GHz, and 12–18 GHz, respectively. Two antennas are used for transmitting and receiving electromagnetic wave, respectively. In the measurement, the incident angle is 5 degrees and the reflection from a metal plate with the same size as the samples is firstly used for normalization. The absorption is then calculated by *A* = 1 − |*S*_11_|^2^, where *S*_11_ is the measured reflection coefficient from the sample backed by a metal plate.
